# Phytochemical composition and pharmacological activities of *Teucrium polium* L. collected from eastern Turkey

**DOI:** 10.3906/kim-2107-13

**Published:** 2021-11-22

**Authors:** Gizem GÜLSOY TOPLAN, Fatih GÖGER, Turgut TAŞKIN, Gülay ECEVİT-GENÇ, Ayşe CİVAŞ, Gökalp İŞCAN, Mine KÜRKÇÜOĞLU, Afife MAT, K. Hüsnü Can BAŞER

**Affiliations:** 1 Department of Pharmacognosy, Faculty of Pharmacy, İstanbul University, İstanbul, Turkey; 2 Department of Pharmacognosy, Faculty of Pharmacy, Anadolu University, Eskişehir, Turkey; 3 Department of Pharmacognosy, Faculty of Pharmacy, Marmara University, İstanbul, Turkey; 4 Department of Pharmaceutical Botany, Faculty of Pharmacy, İstanbul University, İstanbul, Turkey; 5 Department of Pharmacy and Pharmaceutical Services, Tuzluca Vocational School, Iğdır University, Iğdir, Turkey; 6 Department of Pharmacognosy, Faculty of Pharmacy, İstinye University, İstanbul, Turkey; 7 Department of Pharmacognosy, Faculty of Pharmacy, Near East University, Lefkoşa, TR. North Cyprus

**Keywords:** *Teucrium*, GC-GC/MS, antimicrobial, antioxidant activity, LC, MS/MS

## Abstract

*Teucrium* species that belong to the family Lamiaceae have been traditionally used for their medicinal properties. *T. polium* is one the most widespread members of the genus for its use in the treatment of several diseases. In this study, the essential oil and phenolic composition of the aerial parts from *T. polium* were assessed by GC-FID, GC/MS, and LC-MS/MS as well as for its total phenolic content. Several extracts such as *n-*hexane, chloroform, methanol, and infusion were prepared and their antimicrobial, antioxidant, and also acetylcholinesterase activities were studied. According to GC/MS results, *β* -caryophyllene (8.8%), *t*-cadinol (6.2%), (*E*)-nerolidol (5%), *α* -cadinol (5.4%), and α-pinene (4.7%) were identified as main constituents of the essential oil. LC MS/MS analysis of the infusion and the methanol extract showed the presence of 15 phenolic compounds. Moreover, the total phenolic content of each sample was also determined and the infusion had the highest percentage of phenolics. To evaluate the antioxidant properties, the samples were tested by using DPPH” free radical scavenging, FRAP, and CUPRAC activity methods. The infusion showed the strongest radical scavenging activity, whereas *n-*hexane and chloroform extracts exhibited considerable reducing power effects. The MIC values for all of the examined microorganisms ranged from 15 to 2000 μg/mL with respect to antimicrobial activities.

## 1. Introduction

Plants have been used for a variety of different purposes throughout the history of mankind. They are used not only totreat several diseases but also for protecting health and wellbeing [1]. Additionally, many aromatic plants are cultivatedas flavoring agents in perfumery, for use in food preservation, and to enhance flavors in various types of food [2,3]. Among these plants, Lamiaceae family, in particular, has gained more attention and taken an important place in traditional medicine. They are utilized in several industries such as food, cosmetics, and pharmaceuticals, thanks to the secondarymetabolites which give them multiple properties [4].

The genus, *Teucrium* L., commonly known as a ‘germander’, is one of the largest members of the Lamiaceae family, and is represented by almost 36 species (with 48 taxa) in Turkey, 17 of which are endemic [5]. They are distributed in the Mediterranean and Southwestern Asian regions. In many countries, they are consumed as herbal teas, spices appetizers, and are also used to treat numerous health problems such as the common cold, pain, rheumatism, and digestive disorders [6].

Previous studies have revealed that *Teucrium* species possess significant pharmacological effects such as antimicrobial, antiinflammatory, antioxidant, antimalarial, antipyretic, antiulcer, antinociceptive, antidiabetes, antihypertensive, and hypolipidemic [7–11]. Moreover, studies on different cancer cell lines have produced promising results for the treatment of cancer [12,13]. Recent investigations on the phytoconstituents of the genus showed the occurrence of various classes of compounds such as iridoids, terpenoids, flavonoids, phenylethanoid glycosides, tannins, phenolic acids, and essential oils (EOs) [14–17]. Among the diterpenoids, particularly those of the clerodane type, have been demonstrated to be widespread in the *Teucrium* genus [18]. However, recent experiments have shown that these compounds may also have toxic effects [19]. Considering the diversity of major compounds, the essential oil composition of *Teucrium* was primarily characterized with mono and sesquiterpenes such as *α*-pinene, *β*-pinene, caryophyllene oxide, *β*-caryophyllene, germacrene D, and bicyclogermacrene [20–23].

When the antioxidant defense mechanisms produced in the human body are insufficient, the production of reactive oxygen species (ROS) increases and causes antioxidant stress. Many studies have shown that the proliferation of free radicals and oxidative stress are found to be effective in the initiation and progression of many serious illnesses, particularly cancer and neurodegenerative diseases [24]. Alzheimer’s disease is the most common neurodegenerative disorder, correlated with the existence of oxidative stress, a loss of equilibrium between the development and spread of reactive oxygen species, both of which can result in neuronal damage [25].

The widespread interest and usage of plants for their antioxidant, antiaging, and antimicrobial benefits have increased dramatically throughout the world over the last few years. Therefore, many researchers have directed their attention to the pharmacological benefits of the plant kingdom. It has been clearly demonstrated that secondary metabolites of plants, notably essential oils, and phenolic compounds, have heightened benefits in reducing oxidative damage and protecting against the harmful effect of free radicals. Furthermore, awareness of microorganisms’ increased resistance to commercial antibiotics makes plant-sourced derivatives unique because of the efficacious potential in order to develop new drugs [26]. Apart from their importance in medicine, different types of industries turn their attention to the use of natural compounds to avoid degradation and microbial contamination instead of synthetic preservatives. Butylated hydroxytoluene (BHT) and butylated hydroxyanisole (BHA) are the most widely used synthetic antioxidants in industries, yet their long-term exposure revealed the occurrence of adverse effects [2].

*Teucrium polium* L. (felty germander), named as ‘tüylü kısamahmut otu’ in Turkish, is widely distributed particularly in the eastern part of Turkey and its infusions are used in Anatolian folk medicine to treat diabetes, stomach problems, and abdominal cramps [27]. It is still used by local people for several diseases in Ağrı (eastern part of Turkey). To the best of our knowledge, this is the first study on the species growing naturally in this region. The present study is conducted to evaluate its biological potential with phytochemical composition together and contribute to the knowledge of *Teucrium* literature.

## 2. Materials and methods

### 2.1. Plant material

The aerial parts of *T. polium* were collected in Ağrı (Doğubeyazıt), 2017, from an eastern region of Turkey, during the flowering stage. The botanical identification of the collected sample was done and voucher specimens were stored at the Herbarium of the Pharmacy Faculty of İstanbul University (ISTE Number: 116565). The aerial parts were dried at room temperature and kept in a dark place.

### 2.2. Preparation of extracts

The oil was extracted from aerial sections of the plant by hydrodistillation for 3 h with a Clevenger-type apparatus, following Pharmacopoeia’s procedure [28]. The essential oil was maintained at +4 °C in an amber-colored vial until analysis.

The aerial parts of *T. polium* were powdered using a mill and then extracted with different solvents in the order of *n-*hexane, chloroform, and methanol using a Soxhlet apparatus. Thereafter, extracts were filtered through a Whatman paper and evaporated to dryness under reduced pressure at a temperature below 40 °C. Additionally, infusion of the plant was also prepared via the maceration procedure. Ten g of air-dried *T. polium* was macerated by shaking using 100 mL hot water twice and then lyophilized and stored at −20 °C until analysis.

### 2.3. Chemical analyses of essential oil

The oil was examined by capillary Gas Chromatography (GC) and Gas Chromatography-Mass Chromatography (GC/MS) using an Agilent GC–MSD system. The GC analysis was carried out by an Agilent 6890N GC system. The GC/MS analysis was performed with an Agilent 5975 GC/MSD system (Agilent Technologies Inc., Santa Clara, CA). To obtain the same elution order with GC/MS, the simultaneous injection was accomplished by using the same column and operational parameters. In the analysis, the Innowax FSC column (Hewlett-Packard - HP, U.S.A.) (60 m × 0.25 mm, 0.25 μm film thickness) was used, and FID temperature was set at 300 °C. Helium was used as carrier gas (0.8 mL/min.). The temperature of the GC oven was maintained at 60 °C for 10 min and increased to 220 °C at a rate of 4 °C /min, and kept constant at 220 °C for 10 min and then programmed to 240 °C at a rate of 1 °C /min. The split ratio was adjusted 40:1. The injector temperature was at 250 °C. Mass spectra were taken at 70 eV. Mass range was between *m/z* 35–450.

The constituents of essential oil were identified by comparison of their mass spectra with those in the Baser Library of Essential Oil Constituents, Adams Library, MassFinder Library, Wiley GC/MS Library, and confirmed by comparing their retention indices. The relative retention indices (RRI) were calculated using alkanes as reference points. Relative percentage amounts of the separated compounds were calculated from FID chromatograms. The essential oil sample was analyzed three times.

### 2.4. Determination of phenolics using LC–MS/MS

Phenolic compounds were determined with a Shimadzu HPLC 20A system attached to an Applied Biosystems Q-Trap 3200 LC- MS/MS system. Mass spectrum analyses were performed in the negative ionization mode at a mass range of 150–800 amu. 250 × 4.6 mm, 5 μm, ODS analytical column was chosen at 40 ºC for the chromatographic analysis. UV Chromatograms were taken at 280 and 320 nm. CH_3_OH:H_2_O:CH_2_O_2_ (10:89:1, v/v/v) (solvent A) and CH_3_OH:H_2_O:CH_2_O_2_ (89:10:1, v/v/v) (solvent B) were used for the gradient analysis at flow rate 1 mL/min. The composition of B was increased from 15% to 100% in 40 min.

### 2.5. Total phenolic contents of the samples

The FCR method was used to determine the total phenolic content of four different extracts from the aerial parts of the plant. Briefly, 5 μL extract (5 mg/mL −0.5 mg/mL) was taken and 225 μL of water was added in the tube. Then 5 μL of Folin-Ciocalteu reagent (diluted 1/3 with distilled water) and 15 μL of 2% sodium carbonate solution were added to the combination. Afterward, the combination was allowed to rest at room temperature for two h before the absorbance at 760 nm was recorded against the standard reference. The total phenolic content of the extracts was expressed as mg gallic acid equivalents/g extract [29].

### 2.6. Antioxidant activity of the samples

The antioxidant capacity and free radical scavenging activity of extracts were measured by CUPRAC, DPPH^•^, and FRAP assays.

The antioxidant capacity of the samples was determined using the CUPRAC method. A plate was combined with 1 mL of Cu (II) (1.10–2 M), neocuproine ethanolic solution (7.3.10–3 M), and 1 M NH_4_Ac buffer solution. Extracts 1 mL and 0.1 mL pure EtOH were added to the initial mixture to make the final volume: 4.1 mL. After ten s of vortexing, the absorbance of the solution was measured at 450 nm against a reagent blank. Samples of CUPRAC measurements have been demonstrated as equivalents for Trolox (mM Trolox/mg extract) [30].

The DPPH^•^ method was used to test the capacity of free radical scavenging in four distinct extracts. To summarize, 240 μL of DPPH solution (0.1 mM) was mixed with 10 μL of extracts prepared at various concentrations (5 mg/mL −0.5 mg/mL). Then, the mixture was kept for a further 30 min at room temperature before its usage. The absorbance of the combination was determined in comparison to a standard using a microplate reader set at 517 nm. The experiment was replicated three times and the outcomes were given as ascorbic acid equivalent (mg AaE/g extract) [31].

The FRAP method has been studied for evaluating the ability of ferric reducing of different extracts (5 mg/mL −0.5 mg/mL). In brief, the FRAP reagent (3.8 mL) was mixed with samples (0.2 mL) and 4 min later, the absorbance of the mixture was measured against the reference at 593 nm. The standard curve was prepared using FeSO_4_.7H_2_O and FRAP values of the samples were expressed as an mM Fe^2+^/mg extract [32].

### 2.7. Anticholinesterase activity of the samples

The inhibition of cholinesterases was determined using a 96-well microplate reader based on the method developed by Ellman et al. (1961), with some changes. Tris-HCl buffer (50 mM, pH 8.0) was used to prepare all reagent solutions (daily). Shortly, AChE solution (20 *μ*L) were mixed with 20 *μ*L of the sample and 40 *μ*L of Tris-HCl buffer, and the mixture was stood at room temperature (25 °C) for 10 min. Then, 20 *μ*L of ATChI (50 mM) was mixed into the combination and the total mixture was incubated for 5 min at 25 °C. Then, 100 *μ*L of 20 mM DTNB (containing 1M NaCl and 0.2 M MgCl_2_.6H_2_O) was added to the mixture and its absorbance was read at 412 nm against the reference. Each experiment was conducted in triplicate. As a reference compound, galanthamine was used [33].

### 2.8. Antimicrobial activities of the samples

Anticandidal and antibacterial tests were performed according to partly modified CLSI M27-A2 and M7-A7 reference protocols. Amphotericin-B and Ketoconazole (Sigma-Aldrich) were used as standard antifungal agents while Chloramphenicol and Ampicillin (Sigma-Aldrich) were used as antibacterial. *Candida albicans* ATCC 10231, *Candida tropicalis* NRRL Y-12968, *Candida albicans* ATCC 90028, *Candida tropicalis* ATCC 750, *Candida utilis* NRRL Y-900, *Candida parapsilosis* ATCC 22019, *Candida krusei* ATCC 6258 were used as test strains for anticandidal assay. *Escherichia coli* NRRL B-3008, *Staphylococcus aureus* ATCC 6538, *Pseudomonas aeruginosa* ATCC 7853, *Salmonella typhimurium* ATCC 13311, *Serratia marcescens* NRRL B-2544, *Klebsiella pneumonia* NCTC 9633 were used for antibacterial susceptibility test.

Different from the standard protocol, EO and extracts of *T. polium* were diluted between the concentrations of 2 mg/mL to 0.004 mg/mL where the standard antifungals were diluted following CLSI methods [34–35]. Stored yeast strains were refreshed onto potato dextrose agar (PDA, Fluka) while bacteria were inoculated onto Mueller Hinton Agar (MHA, Fluka) for checking purity. All tests were achieved by using sterile 96 U-shaped multi-well plates (Brand). Antimicrobial test results were screened after the incubation period at 35 ±2 °C, 16–20 h. The MIC (minimal inhibitory concertation) is defined as the lowest concentration in which an optically clear well is observed. Furthermore, according to the M27-A2 method, recommended MIC limits of two quality control strains [*C. krusei* (ATCC^®^ 6258) and *C. parapsilosis* (ATCC^®^ 22019)] against Amphotericin-B and Ketoconazole were considered for the precision and accuracy of the assay [36].

### 2.9. Statistical analysis

Results were expressed as mean ± standard deviations (SD) of three independent and parallel measurements. One-way analysis of variance was performed following ANOVA procedures, and significant differences between means were determined by a Tukey Multiple Comparison test.

## 3. Results and Discussion

### 3.1. The yield of essential oil and extracts

The essential oil of *T. polium* obtained by hydrodistillation was a yellow liquid with a distinctive odor with a yield of 0.2%. Aerial parts of the plant were extracted successively with different solvents and the results of fractions yield were found *n-*hexane extract (0.289 g), chloroform extract (1.97 g), methanol extract (6.03 g), and the infusion (0.271 g), by expressing extractable compounds as (EC)/g of dry weight (DW).

### 3.2. Phytochemical composition

Total phenolic compounds found in different extracts were determined using the FCR method. The results were expressed as mg gallic acid equivalent/g extract. The total phenolic contents in the different extracts from the plant were as follows: infusion (59 mg GAE/g extract) > *n-*hexane (11 mg GAE/g extract) methanol (9 mg GAE/g extract) > chloroform (4 mg GAE/g extract). According to the data obtained, it was observed that infusion exhibited the highest total phenolic contents compared to the other extracts.

### 3.3. GC and GC/MS analyses

The essential oil composition of *T. polium* was determined in triplicate by GC-FID and GC/MS analyses. Sixty-six constituents have been described, accounting for 100% of the total components in the essential oil. The retention indices and percentage composition of compounds are demonstrated in Table 1.

The EOs of *T. polium* collected from the eastern part of Turkey, consisted of high percentages of sesquiterpenes, followed by monoterpenes, and fatty acids. The major compounds of EO were *β*-caryophyllene (8.8%), followed by T-cadinol (6.2%), (*E*)-nerolidol (5%), *α*-cadinol (5.4%), and *α*-pinene (4.7%). None of the percentages of the main constituents was detected over the 10 % range in the EO. Additionally, α-bisabolol (4.4%), *β*-pinene (2.4%), (*Z*)-*β*-farnesene (3.4%), *α*-terpinyl acetate (2.8%), *β*-bisabolene (3.2%), caryophyllene oxide (4.3%) were identified with a range from moderate to low percentages in the EO. One of the most common sesquiterpenes is germacrene D in the EO of *T. polium* which is generally found in high amounts, however in this case it was detected as a minor constituent (0.7%). Two fatty acids, dodecanoic acid (0.7%) and hexadecanoic acid (2.9%), respectively, were detected in fairly low concentrations.

According to previous investigations, *Teucrium* EOs were characterized by sesquiterpenes [37]. The major compounds of the EO of *T. polium*, collected from Jordan, were identified as 8-cedren-13-ol (24.80%), *β*-caryophyllene (8.70%), germacrene D (6.83%), sabinene (5.24%), α-humulene (4.34%), allo-aromadendrene (4.54%), δ-cadinene (3.51%) [37]. Bakari studied the chemical composition of *T. polium* collected from Tunisia and found α-pinene (17.04%), *β*-pinene (12.68%), limonene (6.65%), β-myrcene (6.07%), and germacrene D (5.89%) as main constituents [38]. The major compounds of the EO from *T. polium* growing in Iran were spathulenol (15.06%), β-pinene (11.02%), *β*-myrcene (10.05%), germacrene B (10.11%), germacrene D (8.15%), bicyclogermacrene (8.25%), and linalool (4.02%) [39]. In addition, the EO composition of *T. polium* from different regions of Turkey was investigated by several researchers. *β-*caryophyllene (17.8%), *β*-pinene (18%), *α*-pinene (12%), caryophyllene oxide (10%), myrcene (6.8%), and germacrene D (5.3%) have been reported as main compounds in the EO of *T. polium* growing in Gaziantep (southeast of Turkey) while germacrene D (9.69%), α-farnesene (10.7%), *β*-phellandrene (10.8%), and *(Z)- β-*farnesene (15.5%) were detected in high percentages in the EO of *T.polium* from Ardahan (northeast of Turkey) [40–41]. The EO composition of *T. polium* collected from southwest of Turkey was revealed to contain germacrene D (8.10%), carvacrol (5.41%), and *β*-pinene (4.63%) [42]. Farahbakhsh et al. investigated the EO of *T. polium* collected from southeast of Khuzestan province of Iran (Behbahan city) and determined the major constituents as β-pinene (12.97%), *α*-pinene (6.97%), valerianol (21.44%), (E)-caryophyllene (4.71%), epi-α-bisabolol (9.86%), myrcene (2.19%), limonene (3.45%), carvone (3.85%), cis-verbenol (2.11%), (E)-*β*-farnesene (2.23%), and dehydro-sesquicineole (2.86%) [43]. In another investigation on *T. polium* collected from Algeria (Djelfa), *β*-pinene (13.26%) and limonene (12.65%) were observed as the main constituents in the EO [44]. When comparing our results with previous studies in terms of the EO composition, both are quite similar yet the percentages of compounds showed some differences. Multiple studies have shown the existence of carvacrol in many *Teucrium* EOs however, it was not present in the oil investigated in this study. According to the literature data, *T. polium* has a significant chemical variation in the EOs. Differentiations of the components and their amounts are thought to be attributed to the polymorphic variants and growing conditions such as geographical region, altitude, climate, etc., and also vegetation period [37–44].

### 3.4. LC–MS/MS Results

**Figure 1:**
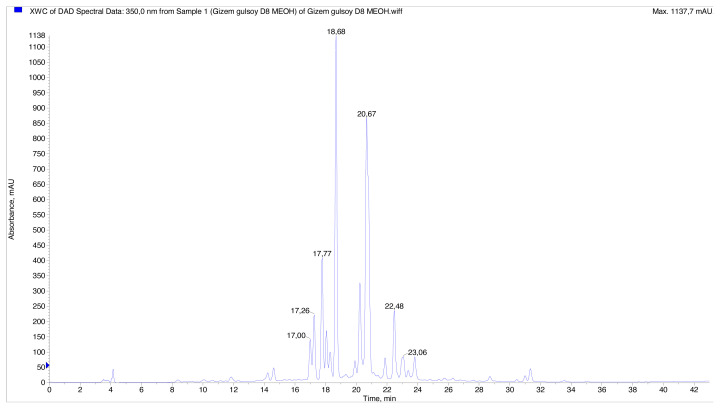
HPLC chromatogram of methanol extract from *Teucrium polium*.

**Figure 2:**
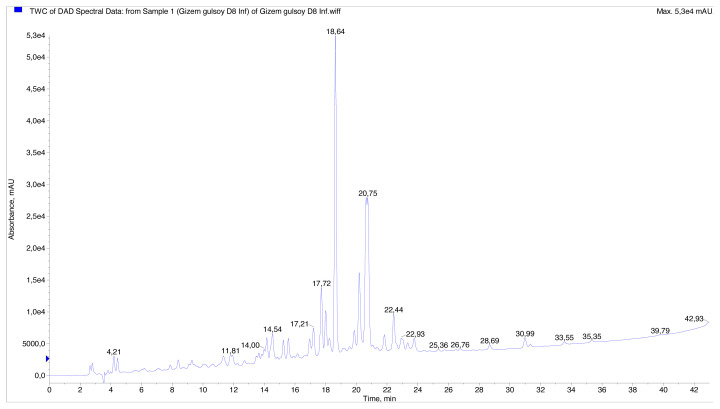
HPLC chromatogram of infusion from *Teucrium polium*.

The presence of 15 phenolic compounds was identified in the infusion and methanol extract prepared from the aerial parts of *T. polium* using LC–MS/MS. The results are demonstrated in Table 2. The method allowed for good separation of the various forms of phenolic compounds found in the samples. The identification of compounds was done using previously published data on *Teucrium* phenolics and chlorogenic acid mass spectrum literature [45–47]. The LC chromatograms of the samples can be seen in Figures 1 and 2.

In the present study, it is observed that the infusion and methanol extracts contain similar phenolics with different quantities. Based on the chemistry of detected compounds, a different class of phenolics was detected such as phenylethanoid glycosides, hydroxycinnamic acid derivatives, flavonoid glycosides, and flavonoid aglycones. Quinic acid, poliumoside, 4-caffeoylquinic acid, and 3-feruloyl quinic acid were in the group of hydroxycinnamic acid derivatives while forsytoside B and verbascoside were identified belonging phenylethanoid glycosides. The most abundant group was flavonoid glycosides which were represented by six compounds, namely vicenin-2, quercetin-glucoside, luteolin-glucoside, luteolin-rutinoside, apigenin-rutinoside, and apigenin glucoside in the samples.

While these compounds were found in varying proportions in both extracts, luteolin glycoside was the major compound. Furthermore, luteolin was identified as the only aglycon in the extracts. Although previous studies have shown the existence of nepetin, cirsilineol, circimaritin in *Teucrium* species, they have not been detected in the analyzed samples.

Several studies have been conducted to reveal the phenolic composition of *Teucrium* species. Decoction and infusion of the aerial parts of *T. polium* were analyzed by LC-MS/MS and luteolin-glycosides, pelargonin, and fumaric acid were exhibited as main constituents [48]. Twelve phenolic compounds were detected in the methanol extract of *T. polium*, containing high levels of catechin, luteolin, apigenin, rutin, quercetin, and chlorogenic acid whereas in the methanolic extracts of *T. scordium* five phenolic compounds were identified such as catechin, rutin, vanillic and *p*-coumaric acids [49]. In another study, the polyphenolic profiles of four *Teucrium* species from Macedonia were investigated and one hydroxycinnamic acid, eight phenylethanoid glycosides, flavonoid aglycones, and glycosides were found in the extract [47]. While the phenylethanoids composed the majority of the identified phenolic substances in Macedonian *T. polium*, flavonoids were the chief group in our study.

When results were compared with previous data, it was observed that the majority of the phenolic compositions of *Teucrium* species consisted of phenylethanoid glycosides and flavonoid derivatives. Although these species contain similar groups of phenolics, a variety could be demonstrated in both different and same species in their chief groups and also components.

According to previous studies, it has been determined that *Teucrium* species contain certain flavonoids and phenolic acids as potential chemical markers and may play a decisive role in the identification of these species [47]. Therefore, the identification of phenolic compounds is significant from the taxonomical and pharmacological points of view.

As a result of our study, valuable phenolics were found in *T. polium* in coherence with the literature. However, some unidentified substances have also been detected. Further studies are required to evaluate these compounds to elucidate their structures and their biological activities.

### 3.5. The results of antioxidant activity

To evaluate the antioxidant capacity of the extracts, it is better to use several assays not only to understand the action of different mechanisms but also to make a comprehensive assessment. In this study, three distinct assays were used to observe the antioxidant capacity of the samples. The results are demonstrated in Table 3.

The DPPH radical scavenging activities of the extracts were determined using the DPPH method. According to the results obtained, the infusion (59.5 mg AaE/g extract) exhibited strong radical scavenging activity compared to the other extracts. In addition, methanolic extracts of the plant did not show DPPH radical scavenging activity.

The iron (III) ion reducing the power of the plant is very important in evaluating their antioxidant potential. The iron reduction power is based on the reduction of the herbal extract Fe^3+^ to Fe^2+^ and measured spectrometrically at 593 nm. According to this approach, a high absorbance indicated that the sample had a high iron reduction potential. This study aims to explore the antioxidant properties of iron (III) ion reduction of various extracts from the plant comparatively. It was determined that *n-*hexane (0.219 mMFe^2+^/mg extract) and chloroform extracts (0.119 mMFe^2+^/mg extract) had stronger iron (III) ion reducing power than other extracts. All the extracts were found to have lower FRAP values than the BHT compound (1.10 mMFe^2+^/mg extract). The Copper (II) ion-reducing antioxidant capacity of different extracts prepared from the plant was evaluated by the CUPRAC method. According to the results, the infusion (0.101 mMTE/mg extract) and *n-*hexane (0.099 mMTE/mg extract) extracts exhibited the strongest copper (II) ion-reducing antioxidant capacity. These findings show that all the extracts were found to have lower CUPRAC values than the BHA compound (1.622 mMTE/mg extract).

According to several studies, the extracts of *T. polium* showed considerable antioxidant potential [48, 50–53]. It is quite well-known that extracts prepared with various solvents possess differing polarities and therefore, show different biological activities as they contain different secondary metabolites. Phenolic compounds are significant secondary metabolites in many plants. One of the largest candidates of these classes is flavonoids which exhibit great antioxidant and chelating properties depending on the chemical structure. Many studies have shown that the existence and also the position of hydroxyl groups are related to their antioxidant capacity [17]. According to LC/MS results, the presence of different flavonoids was identified and antioxidant properties may be linked to the existence of these components in the plant.

### 3.6. Inhibition potential of anticholinesterase

The acetylcholinesterase enzyme inhibition potential of different extracts and galantamine at 500 μg/mL concentration were analyzed comparatively using the Ellman method as shown in Table 4. It was found that the enzyme inhibition potential of infusion (84.381%) and chloroform (64.381%) extracts was higher compared to the other extracts. According to the findings obtained in this experiment, infusion of the aerial parts of the plant showed almost close enzyme inhibition with galantamine (94.52%). On the other hand, it was determined that the methanolic extract had no acetylcholinesterase enzyme inhibition potential at the specified concentration.

Currently, inhibition of the acetylcholinesterase enzyme is a significant way to discover the effectiveness of compounds that might have the potential to be utilized in neurodegenerative disorders. Despite the increasing prevalence of Alzheimer’s disease, no effective treatment has yet been discovered. In Anatolian folk medicine, Ottoman herbalists had suggested the usage of *T. polium* for memory-augmenting performance [54]. In many previous studies, *Teucrium* species have been established as potential inhibitors of the key enzymes in progressive degenerative diseases [9, 55].

To sum up our findings, the infusion of *T. polium* collected in Ağrı is thought to be a promising solution for the inhibition of the acetylcholinesterase enzyme. Responsible compounds should be identified by further investigations on the plant.

### 3.7. The results of antibacterial and anticandidal activities

The antibacterial and antifungal activity of EO and extracts obtained from *T. polium* were studied against 6 bacteria and 7 yeasts using broth dilution methods. Tables 5 and 6 show the MIC results for all samples and standard agents. *T. polium* extracts demonstrated “strong to moderate” inhibitory effects on tested *Candida* and pathogenic bacteria panel between the concentrations of 15 to 2000 μg/mL and 31 to 2000 μg/mL, respectively. Among them, *n-*hexane and chloroform extracts showed better antifungal effects, especially on *Candida tropicalis* and *Candida utilis* at concentrations of 15 and 31 μg/mL, respectively. Methanolic extract showed remarkable antibacterial effects on *S. aureus* having a MIC value of 31 μg/mL. Interestingly, investigated essential oil showed quite a weak activity against all tested microorganisms.

According to our results, especially apolar extracts showed remarkable antifungal activity. As it is seen in Table 6, Gram (+) bacteria *S. aureus* were more susceptible to all extracts while none of them showed strong activity against Gram (−) pathogens. Our findings have confirmed the previous studies that both extracts and EO of *T. polium* collected from different locations had already shown strong inhibitor activity against *S. aureus* [15, 56–57]. El Atki et al. (2020) tested the EO of *T. polium* collected from Morocco against Gram-negative bacteria (*Acinetobacter baumannii, Citrobacter koseri*, *Escherichia coli*, *Pseudomonas aeruginosa*, and *Klebsiella pneumoniae*) and Gram-positive bacteria *S. aureus* and demonstrated that the EO showed notable antimicrobial activity with 15 to 562 μg/mL MIC values. Among the tested bacteria, *A. baumannii* and *S. aureus* were the most susceptible strains of all oil samples analyzed [58]. In another study, EO, ethanol, and aqueous extracts of the aerial parts of *T. polium* subsp. *gabesianum* from Tunisia were examined for antimicrobial potential and showed promising effects especially against *S. aureus* and *Citrobacter freundei* [59].

Numerous studies have shown that the EO and extracts of *T. polium* exhibited remarkable antimicrobial activity which is also supported by our findings [39, 60–62]. Consequently, it may be suggested that further studies are warranted to test extensively the antimicrobial activity against other pathogens and additionally to reveal the responsible components.

## 4. Conclusion

This study has allowed the identification of EO and phytochemical components of *T. polium*, and has demonstrated the effectiveness of biological potential. The characterization of EO, infusion, and methanolic extract was achieved as contributing to the knowledge of their chemotaxonomy. Mono- and sesquiterpene- rich EOs were detected and differences were exhibited compared with the previous studies. The high amounts of phenolic compounds in the infusion showed remarkable anticholinesterase and antioxidant properties. As to the MIC values, the EO and extracts had mild to poor inhibitory effects. Consequently, these findings take into account that *T. polium* extracts could be utilized for pharmaceutical purposes. Further research is needed to assess the functions of other compounds in various parts of the plant.

## Figures and Tables

**Table 1:** The essential oil composition of the aerial parts from *Teucrium polium*.

RRI ^a^	RRI ^b^	Compounds	%	IM
1032	1008–1039 ^c^	α-pinene	4.7	t_R_, MS
1076	1043–1086 ^c^	camphene	tr	t_R_, MS
1118	1085–1130 ^c^	*β*-pinene	2.4	t_R_, MS
1132	1098–1140 ^c^	sabinene	0.4	t_R_, MS
1138	1109–1137 ^c^	thuja-2,4 (10)-dien	0.4	MS
1174	1140–1175 ^c^	myrcene	1.0	t_R_, MS
1188	1154–1195 ^c^	β-terpinene	0.1	t_R_, MS
1203	1178–1219 ^c^	limonene	1.1	t_R_, MS
1213	1188–1233 ^c^	α-phellandrene	0.1	t_R_, MS
1215	1215^k^	*p*-mentha-1, 3, 6-triene	0.1	MS
1255	1222–1266 ^c^	β-terpinene	0.2	t_R_, MS
1266	1232–1267 ^c^	(E)-β-ocimene	0.1	t_R_, MS
1280	1246–1291 ^c^	*p*-cymene	0.3	t_R_, MS
1290	1261–1300 ^c^	terpinolene	0.1	t_R_, MS
1386	1386 ^e^	octenyl acetate	0.4	MS
1454	1446 ^f^	dimethyl tetradecane	tr	MS
1452	1412–1457 ^c^	*p*-cymenene	0.2	MS
1459	1411–1465 ^c^	1-octen-3-ol	0.2	t_R_, MS
1497	1462–1522 ^c^	α-copaene	1.1	MS
1516	1516 ^d^	theaspirane A	tr	MS
1552	1552 ^d^	theaspirane B	tr	MS
1553	1507–1564 ^c^	linalool	0.6	t_R_, MS
1568	1560–1590 ^c^	*trans*-α-bergamotene	0.6	MS
1568	1568 ^e^	1-Methyl-4-acetyl cyclohex-1-ene	tr	MS
1577	1563–1608 ^c^	α-cedrene	0.3	MS
1586	1545–1590 ^c^	pinocarvone	0.6	MS
1590	1549–1597 ^c^	bornyl acetate	0.4	t_R_, MS
1600	1565–1608 ^c^	β-elemene	0.4	MS
1611	1564–1630 ^c^	terpinen-4-ol	0.4	t_R_, MS
1612	1569–1632 ^c^	β-caryophyllene	8.8	t_R_, MS
1651	1612–1654 ^c^	γ-elemene	1.0	MS
1661	1667 ^f^	sesquisabinene	1.0	t_R_, MS
1664	1643–1671 ^c^	*trans*-pinocarveol	0.8	t_R_, MS
1668	1627–1668 ^c^	(*Z*)-β-farnesene	3.4	MS
1687	1637–1689 ^c^	α-humulene	1.1	t_R_, MS
1690	1686 ^d^	α-acoradiene	0.2	MS
1704	1682–1704 ^c^	γ-curcumene	1.7	MS
1707	1672–1718 ^c^	α-terpinyl acetate	2.8	t_R_, MS
1725	1696–1735 ^c^	verbenone	0.2	t_R_, MS
1726	1676–1726 ^c^	germacrene D	0.7	MS
1737	1698–1748 ^c^	β-bisabolene	3.2	t_R_, MS
1755	1711–1756 ^c^	β-curcumene	2.7	MS
1772	1722–1774 ^c^	δ-cadinene	1.1	t_R_, MS
1776	1735–1782 ^c^	γ-cadinene	1.1	MS
1786	1743–1788 ^c^	*ar*-curcumene	0.4	MS
1797	1743–1808 ^c^	myrtenol	0.4	MS
1845	1805–1850 ^c^	*trans*-carveol	0.3	t_R_, MS
1853	1778–1854 ^c^	germacrene B	1.6	MS
1864	1813–1865 ^c^	*p*-cymen-8-ol	tr	t_R_, MS
2000	1980–2092 ^c^	*trans*-sesquisabinene hydrate	2.3	MS
2008	1936–2023 ^c^	caryophyllene oxide	4.3	t_R_, MS
2041	1995–2055 ^c^	(*E*)-nerolidol	5.0	t_R_, MS
2096	2075–2088 ^c^	*cis*-sesquisabinene hydrate	0.6	MS
2170	2090–2189 ^c^	β-bisabolol	4.4	MS
2191	2136–2200 ^c^	T-cadinol	6.2	MS
2214	2214 ^d^	*ar*-turmerol	0.5	MS
2232	2178–2234 ^c^	α-bisabolol	0.6	MS
2246	2186–2250 ^c^	α-Eudesmol	0.3	MS
2255	2180–2255 ^c^	α-cadinol	5.4	t_R_, MS
2256	2146–2256 ^c^	cadalene	tr	MS
2287	2287 ^h^	mustakone	0.6	MS
2300	2275 ^g^	cryptomerione	0.5	MS
2500	2500 ^d^	pentacosane	0.5	MS
2503	2442–2524 ^c^	dodecanoic acid	0.7	t_R_, MS
2622	2510–2633 ^c^	phytol	1.5	MS
2931	2862–2945 ^c^	hexadecanoic acid	2.9	MS
				
		Monoterpene hydrocarbons – MH-MTH	**11.2**	
		Oxygenated monoterpenes - OM	3.3	
		Sesquiterpene hydrocarbons - ST	30.4	
		Oxygenated sesquiterpenes - OST	30.7	
		Diterpenes - DT	1.5	
		Others - D	7.9	
		Total %		
		**Identified compound**	**66**	
		**Total %**	**85**	

RRI^a^; Relative retention indices calculated against n-alkanes. %; calculated from the FID chromatograms. RRI^b^: RRI from literature (c [63], d [64], e [65], f [23], g [66], h [67], k [68]) for polar column values; tr; Trace (<0.1 %). Identification method (IM): t_R_, identification based on the retention times (t_R_) of genuine compounds on the HP Innowax column; MS, identified on the basis of computer matching of the mass spectra with those of the in-house Baser Library of Essential Oil Constituents, Adams, MassFinder and Wiley libraries and comparison with literature data.

**Table 2:** The infusion and methanol extract composition of *Teucrium polium*.

R*_t_*	[M-H]^−^	MS^2^	Identified as
8.0	191	173,145,129, 115	Quinic acid
9.2	535	489, 325, 179,163	Unknown caffeic acid derivative
11.8	353	191, 179, 173	4-Caffeoylquinic acid
11.9	367	193,173, 133	3-Feruloylquinic acid
14.3	405	345, 179, 165	Unknown
14.7	593	503, 473, 395, 383, 353, 325, 297	Vicenin-2
16.3	371	285, 231,121	Unknown
17.3	755	623, 593, 461, 179, 161, 135	Forsytoside B
17.8	623	461, 315, 297, 179, 161, 135	Verbascoside
18.5	463	299/300, 283, 255	Quercetin-glucoside
18.6	769	607, 461, 179, 161, 133	Poliumoside
19.7	611	431, 413, 197,153	Unknown
20.6	447	284, 256	Luteolin glucoside
20.8	593	447, 285,	Luteolin rutinoside
21.9	723	269	Apigenin derivative
22.6	575	269	Apigenin rutinoside
23.1	431	269	Apigenin glucoside
28.8	285	241, 151, 133	Luteolin

* R*_t_*: Retention time

**Table 3:** Antioxidant activity of samples from *Teucrium polium*.

Samples	DPPH (mg AaE/g extract)	CUPRAC(mMtrolox/mg extract)	FRAP assay (mM Fe^2+^/mg extract)
*n*-hexane	17.2 ± 0.9	**0.101 ± 0.003**	**0.219 ± 0.016**
chloroform	9 ± 0.9	0.070 ± 0.004	**0.119 ± 0.010**
methanol	-	0.057 ± 0.003	0.053 ± 0.008
infusion	**59.5 ± 0.4**	**0.099 ± 0.002**	0.027 ± 0.004
BHT			1.1 ± 0.12
BHA		1.622 ± 0.12	

Values are mean of triplicate determination (n = 3) ± standard deviation; a P < 0.05 compared with the positive control, b P < 0.01 compared with positive control, c P < 0.001 compared with positive control.

**Table 4:** Anticholinesterase activity of the extracts from *Teucrium polium*.

Samples	Enzyme inhibition (%) (500 μg/mL)
*n-*hexane	18.857 ± 1.333
chloroform	64.381 ± 1.512
methanol	-
infusion	84.381 ± 0.660
Galantamine	94.52 ± 0.14

Values are mean of triplicate determination (n = 3) ±standard deviation;

* P < 0.05 compared with the positive control,

** P < 0.01 compared with positive control.

**Table 5:** Anticandidal effects of *Teucrium polim* extracts (MIC, μg/mL).

*Candida* panel	Strain no	*n-*hexane	chloroform	methanol	infusion	EO	Std-1	Std-2
*C. albicans*	ATCC 10231	250	250	250	2000	500	0.25	0.06
*C. albicans*	ATCC 90028*	500	500	125	>2000	500	0.5	0.03
*C. tropicalis*	NRRL Y-12968	250	250	250	2000	250	0.25	0.03
*C. tropicalis*	ATCC 750	15	62.5	125	1000	500	0.25	0.03
*C. utilis*	NRRL Y-900	31	62.5	125	500	500	0.06	0.06
*C. parapsilosis*	ATCC 22019	250	500	125	500	500	0.25	0.03
*C. krusei*	ATCC 6258	250	500	500	>2000	250	0.5	0.06

St-1: Amphotericin-B, St-2: Ketoconazole

**Table 6:** Antibacterial effects of *Teucrium polim* extracts (MIC, μg/mL).

Bacteria panel	Strain no	*n-*hexane	chloroform	methanol	infusion	EO	Std-3	Std-4
*Escherichia coli*	NRRL B-3008	>2000	500	>200	>2000	1000	2	1
*Staphylococcus aureus* (+)	ATCC 6538	500	125	31	500	125	0.1	0.5
*Pseudomonas aeruginosa*	ATCC 27853	>2000	>2000	>2000	>2000	>2000	64	32
*Salmonella typhimurium*	ATCC 13311	500	500	500	2000	500	1	1
*Serratia marcescens*	NRRL B-2544	500	500	500	2000	500	32	8
*Klebsiella pneumoniae*	NCTC 9633	500	500	1000	>2000	500	0.5	2

Std-3: Ampicillin, Std-4: Chloramphenicol
